# Immediate admission to the surgery hospital significantly optimises quality indicators in older patients with hip fractures: A before-and-after study

**DOI:** 10.1016/j.tjfa.2025.100014

**Published:** 2025-03-28

**Authors:** José Luis Dinamarca-Montecinos, Alejandra Vásquez Leiva, Carmelinda Ruggiero, Yasna Fernández Barrera, Rayén Gac Delgado, Ada Carrillo, Gedeón Améstica Lazcano, Daniel Vásquez Ulloa, Fernando Aranda, Andrés Pizarro Canales, Graciela Mardones, Constanza Gherardelli Morales, Victoria Novik Assael, Osvaldo Sepúlveda, Jossie Acuña, Carola Aravena Arancibia, Julio Ibarra, Jack Bell, Emma Sutton

**Affiliations:** a University of Valparaíso, Chile. Head of the Orthogeriatrics Program, Adult Orthopedics and Traumatology Service, Dr. Gustavo Fricke Hospital, Viña del Mar, Chile. Chair, Scientific Committee of the Fragility Fracture Network, Chile; b Nutrition and Dietetics Degree, Andrés Bello University, Viña del Mar campus, Chile; c University of Perugia, Department of Medicine and Surgery, Section of Gerontology and Geriatrics, Italy. President, the Fragility Fracture Network Italy; d Department of Care Management, Sub Directorate of Administrative Management, Viña del Mar–Quillota Health Service, Chile; e Department of Emergency and Disaster Management, Viña del Mar–Quillota Health Service, Chile; f Bed management, Care Management Sub Directorate, Viña del Mar – Quillota Health Service, Chile; g Adult Orthopedics and Traumatology Service, Dr. Gustavo Fricke Hospital, Viña del Mar, Chile; h Anesthesia Unit and Surgical Wards, Dr. Gustavo Fricke Hospital, Viña del Mar, Chile; i Adult Emergency Unit, Dr. Gustavo Fricke Hospital, Viña del Mar, Chile. University of Valparaíso, Chile; j Bed Management Department, Medical Sub Directorate, Dr. Gustavo Fricke Hospital, Viña del Mar, Chile; k Faculty of Medicine, University of Valparaíso, Chile, Faculty of Medicine, Andrés Bello University, Chile; l Adult Orthopedics and Traumatology Service, Quilpué Hospital, Chile; m The Prince Charles Hospital. The University of Queensland, Australia; n University Hospitals Birmingham NHS Foundation Trust, Institute of Translational Medicine, Birmingham, United Kingdom. University of Birmingham, School of Nursing and Midwifery, College of medical and Dental Sciences, Edgbaston, Birmingham, United Kingdom

**Keywords:** Hip fractures, Orthogeriatrics, Health policy, Quality management, Protocol

## Abstract

**Background:**

Hip fractures generate high biomedical, social, functional, organisational, and economic costs. There are various quality indicators to guide its management. One of them is surgery within 48–72 h. In Chilean public health system, this indicator has out-of-standard results. This situation could have organizational causes: after hip fracture diagnosis, many older patients are first referred to general hospitals, whilst waiting an orthopedic surgical bed.

**Objective:**

To evaluate the effects of a protocol of immediate-admission to the surgery hospital on organisational and economic indicators of hip-fractured older patients.

**Design:**

Before-and-after study, between 01/01/2017–09/30/2019; 12 months before and 21 months after implementation.

**Setting:**

Regional surgical hospital responsible for 87 % of the older population in its assigned territory, in the more aged region of Chile.

**Participants:**

Anonymised data of 902 hip-fractured older adults (≥ 60 years).

**Intervention:**

Implementation of a protocol that requires immediate admission to the surgical hospital of all older hip-fractured patients at the time of diagnosis.

**Measurements:**

Number of hip-fractured patients with no immediate admission, time to surgery, total in-hospital time, and economic costs. Normality tests (Kolmogorov-Smirnov), non-parametric tests (Chi-squared), Mann-Whitney and Kruskal-Wallis tests were performed. Measures of central tendency (medians and percentiles) were used.

**Results:**

After protocol there was a significant reduction in the proportion of patients referred to general hospitals in both, first and second year (pre=37,8 %; post 1 = 27,3 %; post 2 = 23,3 %, *p* = 0,000). Time to surgery was also significantly reduced (medians bed days pre=15, post 1 = 11, post 2 = 10, *p* = 0,000). Total in-hospital time decreased 21 % (3395 bed days), and there was also a significant decrease in costs from USD130,000 to USD35,000 (*p* = 0,000).

**Conclusion:**

Immediate admission to orthopedic surgical hospital of older adults with hip fractures significantly decreases inter-hospital transfers, time to surgery, total in-hospital time, and direct hospital costs.

## 1 Background

Hip fracture (HF) is a geriatric syndrome with enormous clinical, social, and economic impact [[1], [2], [3]]. Its importance is such that the world has been sectored according to the risk of suffering them, in geographical areas of low, medium, and high risk [4]. From an economic point of view, the costs associated with its occurrence are comparable to the sum of the costs of cardiovascular diseases [2].

In Latin America, hand in hand with the population aging, they have had a significant increase [[4], [5], [6], [7], [8]]. This has revealed important shortcomings in health services, especially public ones, which serve most Latin Americans aged 60 or over [9].

One of these shortcomings is considering this geriatric syndrome as a tributary of a single specialty (Orthopedics), despite being a medical-surgical complication whose standard management in the developed world is carried out in a mixed way between Geriatrics and Orthopedics. The need for co-management between both specialties gave rise, in the 1960s, to Orthogeriatrics. Research in Orthogeriatrics has generated important standards and indicators associated with the quality of care for older people with HF. Among them, the waiting time to perform surgical treatment should be less than 72 hours [[10], [11], [12], [13]].

In Latin America, however, these waiting times greatly exceed this limit [5,14,15], which generates significant medical (in-hospital complications, loss of independence, death), economic and organisational disorders [[14], [15], [16]].

In turn, this makes the bed day, one key indicator of hospital management, precarious: The economic costs associated with the use of hospital beds skyrocket, both due to the slowness of implementing surgery, and because patients suffer serious complications that, in turn, oblige to prolong the stay even more and use specialised resources.

How to achieve, then, that these patients undergo surgery promptly? Once the diagnosis of HF has been made, it is essential that these patients are admitted immediately to the hospital where surgical management will be performed, avoiding any form of referral to less-complex hospitals [17].

Unfortunately, in Latin American public hospitals, it is common practice to defer admitting these patients to the surgical hospital, referring them to general hospitals (or even to their own home) to ‘wait for a bed’ [14], making orthogeriatric care difficult. General hospitals, which do not have the resources or competencies to manage these patients, are becoming congested, which can lead to inadequate clinical management. For example, large volumes of non-operated fractured patients waiting in ‘reservoirs’, made up of the peripheral hospitals where they have been referred to wait, create demand for organisational resources to be pooled to coordinate their ‘rescue’. Finally, patients who wait in general hospitals or their own homes may then experience a worse health state at the time of admission to the surgical hospital, with severe complications, which postpone or even prevent the surgical resolution of HF.

For this reason, it is essential to achieve the admission of these patients to surgical hospitals at the same moment that HF is diagnosed. Despite the published evidence [[10], [11], [12], [13],^16^,^17^], hospital management teams raise the fear that the waiting times accrued in general hospitals will be transferred with them to the specialty hospital, and that time to surgery would not be improved.

Some districts have indeed responded to this fear by continuing to enact delayed admission (with referral instead to the general hospital or the patient's own home). For example, in the more aged administrative territory [18] and with the highest incidence of HF [19] of the Chilean Public Health (the Viña del Mar-Quillota Health Service, VQHS), older people with HF were not always immediately admitted to the specialist surgical hospital. After the diagnosis of HF in the emergency unit, a significant but indeterminate percentage was referred to general hospitals to wait for a specialty bed for surgery. The referral to general hospitals to wait for a specialty bed was associated with delays in surgical resolution, long hospital stays, and high economic costs.

This produced a crisis of resolution capacity in 2017. The crisis consisted of such a high number of older patients with HF occupying hospital beds waiting to be operated on (both in the surgical hospital and the peripheral ones), that no more trauma patients of any kind could be admitted.

In this context, the Orthogeriatric Program led an interdisciplinary team to develop a specific management model for these patients. Using local registries of HF in older people, since 2012 Orthogeriatrics and Bed Management Nursing identified that the frequency of HF in older people in the VQHS was less than 1/day. Considering that the installed surgical capacity for these patients was around 10 per week, there was no risk of exceeding the admission capacity of the Emergency Unit (the place through which these patients enter the Hospital). It was crucial to contrast these data with the management level (medical subdirector) and the heads of the Emergency Unit, Traumatology and Anesthesiology. With them, it was decided to prioritise for surgery the admitted older patients with HF; and request the creation of a centralised committee that would support the generation of a standard that would guarantee the immediate admission of these patients at the surgical level.

This committee entrusted Orthogeriatrics with the generation of a protocol that included two major aspects:

a. the immediate admission of older patients with HF to a surgical hospital, avoiding referral to general hospitals or home.
b. the proper management of these patients in the Emergency Unit of the corresponding surgical hospital.

The first measure involved coordination with local authorities at the organisational level and working with general practitioners from the emergency units of all peripheral hospitals, especially in terms of rejecting referrals of these patients to their centres.

The second measure involved working with medical specialties and heads (Anesthesiology, Traumatology, Internal Medicine, Infectology, Cardiology, and Endocrinology) and Nursing (Bed Management) to support interdisciplinary measures to be implemented upon admission of these patients and facilitate their prompt surgery.

Once the final version of the pProtocol was drafted, it was approved as a regional regulation by the director of the health service [20]. Subsequently, it moved on to the implementation phase, also under the responsibility of Orthogeriatrics, which had the support of the regulation, the central commission, and the medical and nursing heads.

The protocol was implemented starting January 2018, and our principal aim with this work is to evaluate the impact that the protocol measure ‘immediate admission to the surgical hospital of older patients with HF’ had on direct surgical hospital admission, time to surgery from initial fracture, total in-hospital stay times, and economic costs. On the other hand, we try to determine if there are differences in the way of admission depending on the origin of the patients.

We set out to carry out this work because in many parts of the world with emerging economies, the epidemiological and organisational realities are similar, and it is useful to know what solutions have been effective in optimising the quality of health systems and approaching compliance with standards. On the other hand, since the administrative territory in which these measures were implemented is the more aged in the country, we think that it is possible that many of the phenomena that currently occur there, will be replicated in other Chilean health services, as their population ages. Finally, even though the standards to be achieved in Orthogeriatrics are clear, we did not find publications that described whether models like ours could be useful to meet those standards.

This work seeks to demonstrate the harmfulness of a common behavior among physicians in countries with developing economies: deferring the admission to the surgical hospital of older patients with HFx. For this reason, we hope that it will be read with interest and that it will contribute to changes in the above behaviors.

## 2 Methods and materials

### 2.1 Aims

The primary aim of the study is to evaluate the impact that the protocol intervention "immediate admission to the surgical hospital of older patients with hip fractures" had on direct surgical hospital admission, time to surgery from initial fracture, total in-hospital stay times, and economic costs. The secondary aim is to determine if there are differences in the way of admission depending on the origin of the patients.

### 2.2 Study design

This is a cross sectional study comparing data before and after the implementation of a protocol intervention in large administrative territory of the Viña del Mar – Quillota Health Service (VQHS). The VQHS is responsible for health care in 18 municipalities in the Valparaíso region of Chile. During the study period, all HF patients from the 18 municipalities were treated at the Hospital Gustavo Fricke (HGF).

**Fig. 1:**
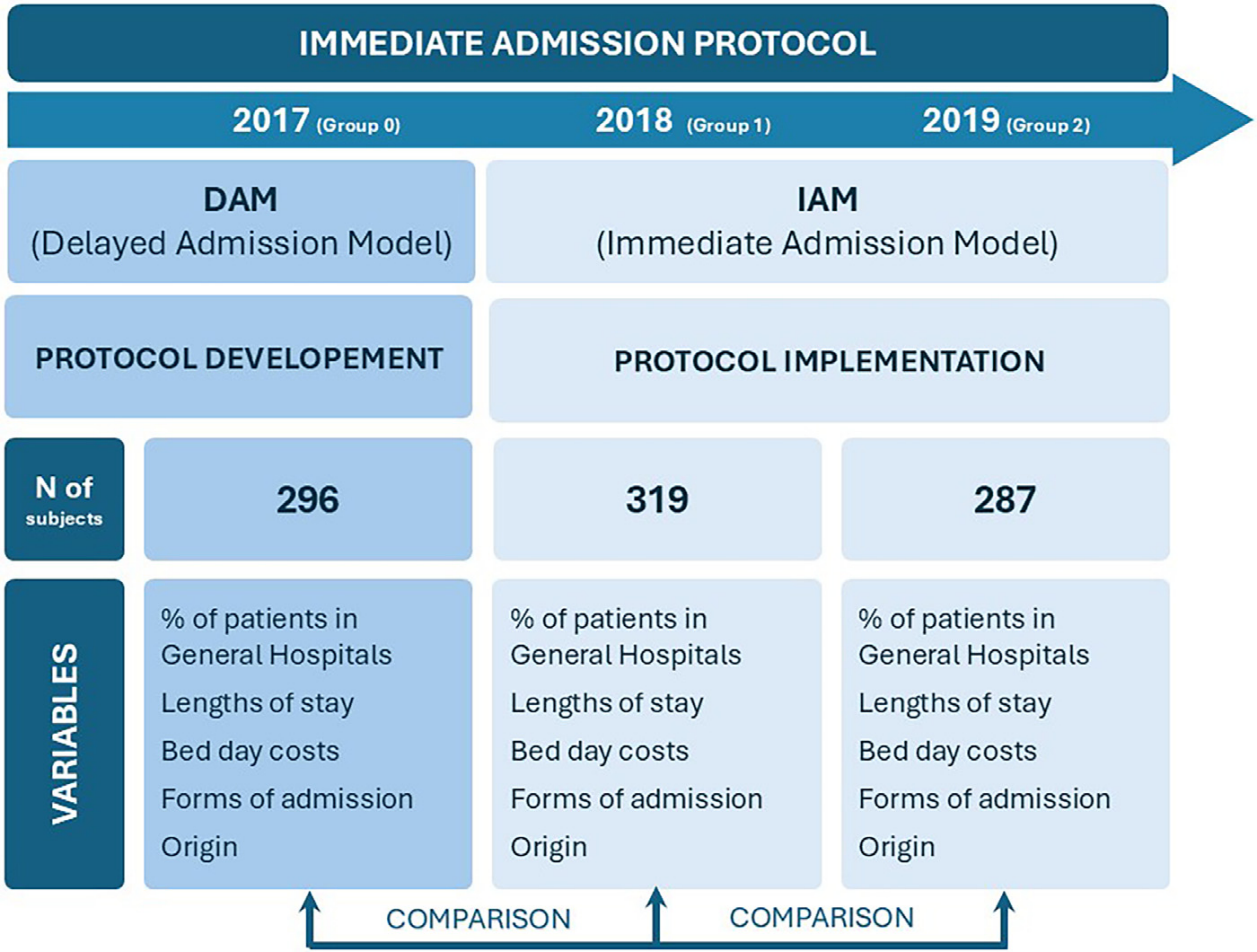
Design Scheme and Time-Flow.

In details, data corresponding to 2017 (year without application of immediate admission measures) were compared with 2018 and 2019 (both years with application of immediate admission), seeking to establish whether there was significance in the impact of outcome measures (See Fig. 1).

On October 18th, 2019, an important social and political crisis broke out in Chile (the "Social Outbreak"), seriously affecting public services and hospitals. Among other things, the incidence of HF decreased until March 2020, when it recovered for a couple of months, before falling again, due to the COVID-19 Pandemic. Surgical waiting times in older people have increased because of the occurrence of trauma injuries among young people, due to fights between rival factions, punishments by the police, assaults, etc. On the other hand, during the Pandemic years (2020 and 2021), HF surgery in older people ceased to be a priority for hospital managers, and we had to defend what had been achieved up to that moment. For these reasons, we think that including data from those periods in this work will be an important source of bias for its objectives and negatively alter the interpretation of the results.

### 2.3 Description of the sample

Anonymised (de-identified and coded) data from the Dr. Gustavo Fricke Hospital (DGFH) discharges, corresponding to the diagnosis of HF, between January 1st, 2017, and September 30th, 2019 (33 months). Both sexes, aged 60 or over. The diagnosis of HF was performed by a Traumatologist-Orthopedist. For purposes of HF diagnosis, the ICD-10 codes S72.0, S72.1 and S72.2 were considered. Anonymised records from the DGFH Orthogeriatrics program database were used to obtain the data of the variables. DGFH belongs to the administrative territory of the Viña del Mar – Quillota Health Service (VQHS). It has the availability of specialised human resources (hip orthopedic surgeons, ortho geriatrician, anesthesiologists), surgical wards, implants, and items necessary for HF surgeries. During the period studied, the entire population dependent on public health in the territory of the VQHS who suffered a HF had to undergo surgery at the DGFH, and there was no alternative public center. This allows working with a complete data collection, equivalent to an *n* of HF over 90.3 % of the population aged 60 or over in the territory studied.

Participants were grouped according to their way of admission, which was defined as delayed or immediate. The delayed admission pathway belongs to subjects who, after the diagnosis of HFx, were referred to a general hospital, waiting to be admitted to the surgical hospital to the delayed group. The immediate admission pathway includes subjects admitted to the surgical hospital during the HFx diagnosis (quality standard). In addition, the subjects were divided according to whether they came from cities near or far from the surgical hospital. For this, the current administrative division of the SSVQ was used as described below. The definition of near cities included Coastal Microgrid (Viña del Mar, Quintero, Concón, and Puchuncaví) and Marga-Marga Microgrid (Quilpué, Villa Alemana, Limache, and Olmué). The definition of distant cities included Quillota-Petorca Microgrid (Quillota, La Cruz, La Calera, Hijuelas, Nogales, Zapallar, Papudo, La Ligua, Cabildo and Petorca).

### 2.4 Data collection

Data were gathered about age, sex of participants. Then, the following dependent variables were considered: the number and annual percentage of hip fractured subjects hospitalised in general hospitals; and the hospital timings, including time to surgery (TTS), time in surgical hospital (TSH), time in general hospitals (TGH), total in-hospital time (TIHT). TTS was defined as the number of days elapsed between the date of HF diagnosis and the date of surgery, both included. This variable is only measurable among subjects who underwent surgical treatment. A separation was included in subjects who underwent surgery in 3 days or less (quality standard), and those who underwent surgery in more than 3 days. TSH was the sum of bed days used since admission to the surgical hospital. The first day counts as day 1, and the day of discharge counts as the last day. TGH was the number of bed days used during hospitalisations in general hospitals, counting the day of admission as day 1, and the date of transfer to the surgical hospital as the last day. TIHT was the sum of all bed days used by each subject, regardless of the hospital of permanence (TIHT=TSH+TGH). The day of hospitalisation counts as day one, and the day of discharge counts as the last day. In those cases where the subject was in more than one hospital, it corresponds to the sum of bed days in all hospitals. Based on the regulations of the Chilean Ministry of Health, the subjects were separated into two groups: prolonged hospitalization (15 or more days) or not (<15 days, quality standard). Economic costs were also estimated as daily costs (38,430 Chilean pesos, equivalent to approximately 52 US dollars at the time) per bed day in VQHS hospitals in the studied period (data provided by the VQHS Accounting Unit).

### 2.5 Statistical analysis

Data were tabulated using an anonymised mask, and consistency analyses were subsequently performed using the SPSS program (Version 20). Normality tests (Kolmogorov-Smirnov) were performed, measures of central tendency (medians and percentiles), and non-parametric tests were used; Mann-Whitney and Kruskal-Wallis tests.

## 3 Results

The sample comprised a total of 902 subjects, with a median age of 83 years. 80.6 % of the subjects were women. There was no significant difference between age and sex between the two groups (*p* < 0,05). Throughout the period, 16,198 bed days were used, with a median of 15 days (P25=9 – P75=23), and an average of 18.3 days per subject. 266 subjects had delayed admission (29.5 %), using 3861 bed days in peripheral hospitals (23.9 % of total bed days). 190 subjects (21.06 %) came from distant cities. Delayed admission occurred in 46 % of the subjects from distant cities and in 25 % of the subjects from near ones. Regarding the waiting time for surgery, the median was 11 days. The medians of hospital times per year can be consulted in Table 1.

**Table 1:** Patients’ characteristics reflecting their management and stratified by groups.

	**Group 0 DAM −2017 (n:296)**	**Group 1 IAM −2018 (n:319)**	**Group 2 IAM −2019 (n:287)**	**P value**
**VARIABLES**				
**Time to surgery**, median (IQR)	15 (11–23)	11 (10–20)	10 (9–18)	<0.001
**Length of stay,** median (IQR)				
- Total	17 (11–21)	13 (6–14)	14 (5–12)	<0.001
- Specialised Hospital	13 (9–17)	11 (7–15)	12 (6–13)	0.009
- General Hospitals	22 (12–24)	7 (6–12)	7 (6–10)	<0.001
**Bed days**, number				
- Total bed days	6567	4955	4676	<0.001
- Bed days, General Hospital	2161	1043	657	<0.001
**Costs** (USD) **associated with bed days**, mean (SD)	1154 (520)	807,7 (312)	847,2 (208)	–
- Total	341,484	257,660	243,152	<0.001
- General Hospital	112,372	54,236	34,164	<0.001
**Patients with delayed admission**, n (%)	112 (37.8)	87 (27.3)	67 (23.2)	<0.001
**Immediate Admission Model (IAM)**				
- TTS, median (IQR)	10 (8–13)	7 (5–10)	6 (3–8)	<0.001
- TTS <3 days, n (%)	21 (7)	29 (9)	34 (12)	<0.001
- LOS-SH, median (IQR)	10 (7–13)	9 (7–11)	9 (6–11)	<0.001
- T-LOS, median (IQR)	10 (7–13)	9 (7–11)	9 (6–11)	<0.001
- T-LOS <15 days, n (%)	163 (55)	207 (65)	195 (68)	<0.001
**Delayed Admission Model (DAM)**				
- TTS, median (IQR)	24 (18–31)	16.5 (15–22)	20.5 (12–23)	<0.001
- TTS <3 days, n (%)	0	0	0	–
- LOS-SH, median (IQR)	30 (14–38)	10 (8–15)	12 (9–15)	<0.001
- T-LOS, median (IQR)	24 (17–32)	18 (13–20)	13 (10–16)	<0.001
- T-LOS <15 days, n (%)	27 (9.1)	96 (30.1)	59 (20.6)	<0.001

Legend. DAM Delayed Admission Model; IAM: Immediate Admission Model; IQR: Inter quartile range, TTS: Time to surgery, LOS-SH: Length of stay in surgical hospital; T-LOS: Total length of stay; USD: US Dollars. Data are expressed as frequencies and percentages or medians and 25th −75th interquartile (IQR), as appropriate. Group 0: pre- intervention with DAM in 2017; Group 1 and 2: post-intervention with IAM in 2018 and 2019, respectively.

Notably, since the implementation of immediate admission, the number, percentage and median of subjects hospitalised in general hospitals decreased markedly and sustainably (*p* = 0.000), and a marked and sustained gradient can be observed in the decrease in time to surgery, reaching a reduction of 5 days in the third year of analysis (*p* = 0.00). Focusing on the time spent in the surgical hospital, since the implementation of the protocol, the time decreased by 3 days (*p* = 0.009), with the biggest impact observed in the first year. The median then stabilises, but the result is still significant. Similarly, a marked and sustained gradient can be observed in the decrease of time in general hospitals (*p* = 0.000) and total in-hospital time with a decrease of 3 days (*p* = 0.00), mainly occurring during the first year. The median then stabilizes, but the result is still significant (Table 1).

**Fig. 2:**
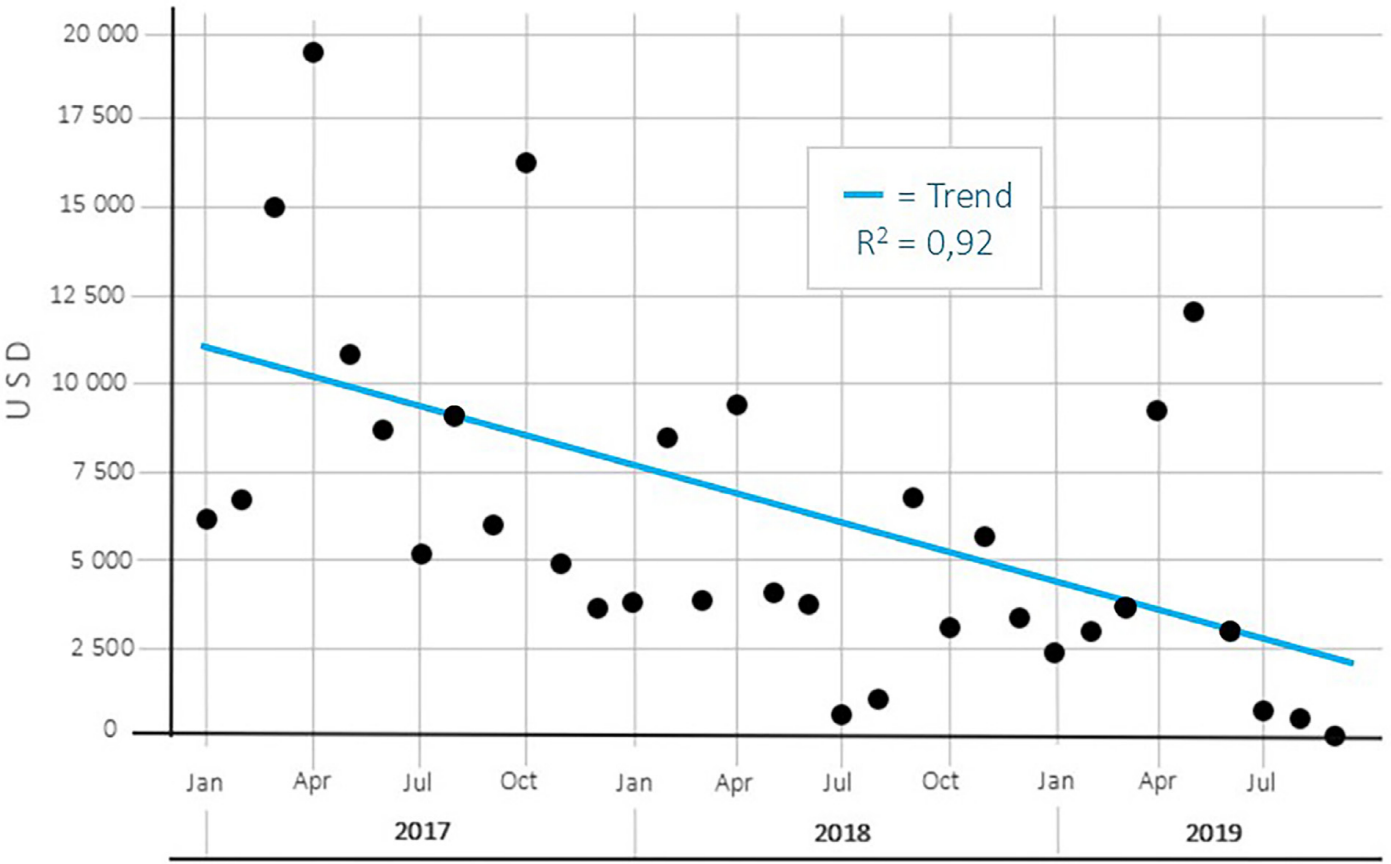
Monthly Evolution and trend of Economic costs associated with bed days in general hospitals (in USD) for older patients with hip fractures, before and after implementing the immediate admission protocol.

After the implementation of immediate admission, the economic costs associated with the hospitalizations of HF patients decreased significantly (*p* = 0.000). In 2017, the cost associated with the use of general hospital beds was around US$110,000. After implementing the protocol, in 2018 it decreased to around USD 55,000, and to around USD 35,000 in 2019. The monthly evolution of this variable can be seen in Fig. 2.

Interestingly, the way of hospital admission drives the hospital times (Table 2). Subjects with delayed admission had a longer time to surgery, more time in the surgical hospital, and longer total hospitalisations than those admitted immediately (*p* = 0.00). When we analyze the evolution year by year (Table 1), we see that time to surgery significantly decreased and remained lower in the immediate admission group; it was always less than that of the deferred admission group (*p* = 0.000). The time in the surgical hospital decreased significantly in the delayed admission group, remaining constant in the immediate admission group. Total in-hospital time remained constant in the immediate admission group, year after year, without significant differences (*p* = 0.87), but was always lower than in the delayed admission group (*p* = 0.000). In the delayed admission group, the total in-hospital time increased year after year (*p* = 0.000) (Table 1).

**Table 2:** Organisational aspects according to participants’ site of origin (panel A) and type of admission (B).

**A)**	**Site of origin**		
	**Distant (n:190, 21 %)**	**Near (n:712, 79 %)**	**p value**
Delayed Admission Model, n (%)	118 (62.1)	153 (21.5)	<0.001
Immediate Admission Model, n (%)	72 (37.9)	559 (78.5)	<0.001
T-LOS, median (IQR)	24 (13–33)	12 (6.5–16)	<0.001
LOS-SH, median (IQR)	15 (10–22.5)	10 (6.5–17)	<0.001
TTS, median (IQR)	20 (11–29)	9 (6–14)	<0.001
**B)**	**Type of admission**		
	**DAM (n:271, 30 %)**	**IAM (n:631, 70 %)**	**P value**
T-LOS, median (IQR)	21 (11–32)	12 (7.5–18)	<0.001
LOS-SH, median (IQR)	17 (10.5–30)	12,5 (7–19)	<0.001
TTS, median (IQR)	30 (16–42)	10 (8–16,5)	<0.001

Legend. DAM Delayed Admission Model; IAM: Immediate Admission Model; IQR: Inter quartile range, TTS: Time to surgery, LOS-SH: Length of stay in surgical hospital; T-LOS: Total length of stay; USD: US Dollars. Data are expressed as frequencies and percentages or medians and 25th −75th interquartile (IQR), as appropriate.

Table 1 also reports the relationship between the way of admission and standards of quality. Surgical patients in the group with delayed admission never underwent surgery within the expected standards (<3 days). On the other hand, in the group of subjects with immediate admission, the percentage of subjects operated on in less than 3 days increased progressively and significantly. The implementation of immediate admission was associated with a significant increase in the percentage of non-prolonged total hospitalisation times (<15 days), both in the group of subjects with deferred admission and in the immediate admission group. In the group with immediate admission, however, the incidence of subjects with hospitalisations of less than 15 days was always significantly higher. Ultimately, Table 2 shows the impact of origin on hospital times. Subjects from distant cities presented significantly longer time to surgery (*p* = 0.000) and longer hospitalisations (*p* = 0.000).

## 4 Discussion

The sample studied presents similar characteristics in terms of gender and age to other published samples [9,10], from countries with emerging economies and/or in advanced epidemiological transition.

In general, scientific literacy gives a static account of the hospital times associated with HF, indicating compliance or not with quality standards. Our results are dynamic, showing that, although hospital times are long compared to the standards [[10], [11], [12], [13]], there is a progressive improvement from implementing the immediate admission model.

The first objective of the protocol regulations was to reduce the number of subjects with delayed admission, and to optimize the surgical opportunity for all patients. This was achieved progressively, significantly decreasing, year by year, the number of patients referred to other hospitals to wait for bed after the diagnosis of HF. Similarly, this meant a progressive increase in the number of patients admitted immediately.

Contrary to the fears of hospital managers, together with the increase in the number of subjects immediately admitted to the surgical hospital, there was a decrease in the days waiting for surgery, without increasing time in the surgical hospital. Indeed, all times related to hospitalisation in the surgical center (time to surgery, time in surgical hospital, and total in-hospital time) showed significant decreases. In addition to the general analysis of the sample, this effect was maintained when analysing the immediate admission subgroup and comparing it with the delayed admission one.

The results show that delayed admission has more late surgeries and longer hospital stays. For its part, immediate admission has shorter preoperative times, a progressively greater number of subjects with preoperative times within international standards, shorter total hospital times, and progressive fewer subjects with prolonged hospitalisations.

This can be explained by two factors. The first is that patients could be immediately identified and incorporated into the pool of patients pending for surgery. This allowed time to surgery to decrease-and-not-increase. Along with this, it has been described that prompt surgery decreases the risk of in-hospital medical complications, which occur mainly in the preoperative phase [[10], [11], [12], [13],^16^].

Time to surgery appears with a median of 15 days during the first year of analysis, prior to the implementation of the Protocol. Since the standard for trading is a maximum of three days [[10], [11], [12], [13]], this results in a *delta* against 12 days. Under the conditions of the present study, immediate admission reported a decrease in excessive time to surgery equivalent to 5 of these 12 days, that is, a little more than 40 %, during a period of 21 months after its implementation. In other words, delayed admission generates time in general hospitals, which is a component of excess preoperative time. Thus, in this sample, time in general hospitals was responsible for about 40 % of the excess time spent waiting for surgery.

In turn, this implies that the impact of the measure has limits, which can be explained as there are many variables of different origin (clinical, economic, political, other organisational variables, etc.) that also influence hospital times.

Regarding the evolution of economic costs, the significant decrease year by year was consistent with the progressive decrease of the two components of this variable that were analysed: length of stay in general hospitals and length of stay in the surgical hospital. Immediate admission proved to be associated with both, causing costs to decrease since the protocol was implemented in January 2018. As the number of deferred patients decreased, the costs associated with days of stay in general hospitals reached zero in the last month of analysis, when no subject entered delayed; and no time increase in the surgical hospital.

Throughout the period, the total expense per bed day was USD 842,296. Of these, USD 200,772 correspond to expenses from time in general hospitals. In other words, almost a quarter of the costs associated with bed days are saved with immediate admission, considering that there was no significant increase in time in surgical hospital. This, without considering more exact standardised management methods for quantifying costs, such as groups related by diagnosis (GRD), which would increase the associated costs, particularly when considering the intrinsic severity of HF.

In relation to the form of admission and the origin, it is interesting to note that the total number of patients who were admitted on a delayed basis was important: close to a third (31.2 %). Of this percentage, close to a third (31.8 %) came from distant cities, accounting for a third (32.9 %) of the total days delayed.

The more favorable times associated with coming from nearby places cannot be explained by chance. Considering that the “distant” communes are rural places, one reason that would explain delaying admission could be a certain type of discrimination due to rurality, which has been described in other publications [21].

This could happen because, when the HF occurs, patients from distant cities are transferred to the surgical hospital "for diagnostic confirmation" and, in many cases, without family members or companions. Once the diagnosis is confirmed, it seems like a "natural" flow (or, at least, "feasible") to defer the patient, using the same ambulance that took him from the peripheral hospital. The reason for the original referral has been fulfilled, and there are no relatives who advocate immediate admission.

While this is a plausible explanation, it does not justify the decision not to enter immediately. This shows the importance that institutional protocols can have in modifying medical behaviours. In this case, by improving quality indicators for dHF patients from distant cities, immediate admission would help does not justify the decision not to enter immediately. This shows the importance that institutional protocols can have in modifying medical behaviours. In this case, by improving quality indicators for HF patients from distant cities, immediate admission would be helping to reduce a form of discrimination, making the health system fairer and more sustainable [22].

The referral to "*wait for bed*" to general hospitals is a phenomenon of unclear causes, which also differ according to the reality of each country. In some cases, it is possible that this is intended to avoid a possible “collapse” of emergency services (due to the increasing incidence of HFx in the region). In others, the lack of knowledge in Orthogeriatrics may lead to the belief that these patients can wait for a delayed surgery without generating major problems. On the other hand, some countries with public systems do not include the implants necessary for these surgeries among their services [14]. This means that these items must be purchased by the patient or relatives, which is not always possible in the short term. The lack of interdisciplinary health teams with knowledge in Orthogeriatrics, geriatrics, traumatology and orthopedics, gerontology, and epidemiology, at the organisational level - with the consequent lack of registries and local action protocols - contributes to exacerbating the problem [23,24]. Finally, these and other causes often coexist. The discrimination by geographical origin would be a new element to consider as an explanation.

Our results show the importance of having specialists in the orthogeriatric area who work together with other specialties and professions around HFx, making the teams aware of the weight of this geriatric syndrome as an acute disease. Finally, it also accounts for the influence that a protocol measure can have on the organisational behavior of daily clinical activity.

## 5 Conclusions

The immediate admission of older patients with HF to a surgical hospital significantly influenced the measured variables, decreasing the time to surgery, total hospital time, time in general hospitals, and economic costs associated with bed days.

There was no carryover effect from the length of stay in general hospitals to the surgical hospital.

The present results advise against the practices of delayed admission of HF older patients, as they have negative organisational and economic effects. Therefore, delayed admission of these patients should be considered unethical.

We suggest implementing measures that guarantee the immediate admission of older patients with hip fractures to the corresponding surgical hospitals.

## Ethics approval and consent to participate

Resolution 06/2024 (May 16th, 2024) of the Scientific Ethics Committee of the Dr. Gustavo Fricke Hospital and Viña del Mar – Quillota Health Service.

## Consent for publication

Not applicable.

## Availability of data and materials

The datasets used and/or analysed during the current study are available from the corresponding author on reasonable request.

## Disclosure statements

José Luis Dinamarca-Montecinos declares that he has no conflicts of interest, and that he has also declared this in the corresponding ICMJE form.

Alejandra Vásquez Leiva declares that she has no conflicts of interest, and that she has also declared this in the corresponding ICMJE form.

Carmelinda Ruggiero declares that she has no conflicts of interest, and that she has also declared this in the corresponding ICMJE form.

Yasna Fernández Barrera declares that she has no conflicts of interest, and that she has also declared this in the corresponding ICMJE form.

Rayén Gac Delgado declares that she has no conflicts of interest, and that she has also declared this in the corresponding ICMJE form.

Ada Carrillo declares that she has no conflicts of interest, and that she has also declared this in the corresponding ICMJE form.

Gedeón Améstica Lazcano declares that he has no conflicts of interest, and that he has also declared this in the corresponding ICMJE form.

Daniel Vásquez Ulloa declares that he has no conflicts of interest, and that he has also declared this in the corresponding ICMJE form.

Fernando Aranda declares that he has no conflicts of interest, and that he has also declared this in the corresponding ICMJE form.

Andrés Pizarro Canales declares that he has no conflicts of interest, and that he has also declared this in the corresponding ICMJE form.

Graciela Mardones declares that she has no conflicts of interest, and that she has also declared this in the corresponding ICMJE form.

Constanza Gherardelli Morales declares that she has no conflicts of interest, and that she has also declared this in the corresponding ICMJE form.

Victoria Novik Assael declares that she has no conflicts of interest, and that she has also declared this in the corresponding ICMJE form.

Osvaldo Sepúlveda declares that he has no conflicts of interest, and that he has also declared this in the corresponding ICMJE form.

Jossie Acuña declares that she has no conflicts of interest, and that she has also declared this in the corresponding ICMJE form.

Carola Aravena Arancibia declares that she has no conflicts of interest, and that she has also declared this in the corresponding ICMJE form.

Julio Ibarra declares that he has no conflicts of interest, and that he has also declared this in the corresponding ICMJE form.

Jack Bell declares that he has no conflicts of interest, and that he has also declared this in the corresponding ICMJE form.

Emma Sutton declares that she has no conflicts of interest, and that she has also declared this in the corresponding ICMJE form.

## CRediT authorship contribution statement

**José Luis Dinamarca-Montecinos:** Conceptualization, Data curation, Formal analysis, Investigation, Methodology, Project administration, Supervision, Validation, Visualization, Writing – original draft, Writing – review & editing. **Alejandra Vásquez Leiva:** Methodology, Formal analysis, Software, Writing – review & editing. **Carmelinda Ruggiero:** Conceptualization, Investigation, Resources, Visualization, Writing – original draft, Writing – review & editing. **Yasna Fernández Barrera:** Conceptualization, Investigation, Writing – review & editing. **Rayén Gac Delgado:** Conceptualization, Investigation, Writing – review & editing. **Ada Carrillo:** Conceptualization, Investigation, Writing – review & editing. **Gedeón Améstica Lazcano:** Conceptualization, Investigation, Writing – review & editing. **Daniel Vásquez Ulloa:** Conceptualization, Investigation, Writing – review & editing. **Fernando Aranda:** Conceptualization, Investigation, Writing – review & editing. **Andrés Pizarro Canales:** Conceptualization, Investigation, Writing – review & editing. **Graciela Mardones:** Conceptualization, Investigation, Writing – review & editing. **Constanza Gherardelli Morales:** Conceptualization, Investigation, Writing – review & editing. **Victoria Novik Assael:** Conceptualization, Investigation, Writing – review & editing. **Osvaldo Sepúlveda:** Conceptualization, Investigation, Writing – review & editing. **Jossie Acuña:** Conceptualization, Investigation, Writing – review & editing. **Carola Aravena Arancibia:** Conceptualization, Investigation, Writing – review & editing. **Julio Ibarra:** Conceptualization, Investigation, Writing – review & editing. **Jack Bell:** Conceptualization, Writing – review & editing. **Emma Sutton:** Conceptualization, Writing – review & editing.

## Declaration of competing interest

The authors declare that they have no competing interests.
